# Opioid Overdose Reversal With Nalmefene Nasal Spray: A Case Report

**DOI:** 10.7759/cureus.85912

**Published:** 2025-06-13

**Authors:** Michel Sucher, Lisa Emmans

**Affiliations:** 1 Substance Use and Addiction, Honor Health Addiction Medicine, Phoenix, USA; 2 Emergency Department, Yavapai Regional Medical Center, Prescott Valley, USA

**Keywords:** emergency responders, nalmefene, opioid overdose, police, substance use disorder

## Abstract

Despite the recent FDA approval of intranasal (IN) nalmefene, there is a lack of published evidence regarding its utilization, namely regarding concerns about its potential for precipitate withdrawal. In this case report, two physician investigators independently reviewed Oakland County (Michigan) Sheriff’s Office (OCSO) bodycam footage, OSCO dispatch report, and the Alliance of Coalitions for Healthy Communities interview data conducted 72 hours post-overdose of a patient who received IN nalmefene following an opioid overdose, to describe clinical signs and symptoms before, during, and after administration. An increase in oxygen saturation up to 98% occurred within approximately 2.5 minutes after the initial dose. The patient also showed a marked improvement in mental status. The patient exhibited no evidence of precipitated withdrawal. This case study provides data to support that IN nalmefene can produce a rapid and effective reversal of opioid overdose. Additional studies will be important in establishing the role of IN nalmefene in the treatment of opioid overdose by first responders as well as its impact on clinical outcomes once patients arrive at the accepting medical facility.

## Introduction

The opioid epidemic continues to evolve in the United States (US), with fentanyl and other synthetic opioids embedded in the illicit drug market. Two opioid antagonists are approved by the US Food and Drug Administration as overdose reversal medications. Naloxone was first approved as an injection in 1971, then as a 4 mg (2015) and 8 mg (2021) intranasal (IN) formulation. Recently, 3 mg and 4 mg IN naloxone formulations were approved for over-the-counter use [1,2]. Parenteral nalmefene (Revex®) was first approved in 1995. In May 2023, IN nalmefene (2.7 mg) was approved to reverse a known or suspected overdose induced by natural or synthetic opioids [3,4]. A nalmefene auto-injector was approved in August 2024.

Data from the State Unintentional Drug Overdose Reporting System database indicates naloxone was administered in over 20% of overdose deaths nationally, with some jurisdictions reporting over 35% [5]. Despite the availability of multiple naloxone products and saturation strategies [6], the estimated death toll from opioid overdoses exceeded 77,000 for the 12-month period ending March 2024, with over 90% due to synthetic opioids [7]. A high-affinity opioid antagonist like nalmefene has the potential to rapidly reverse overdoses involving synthetic opioids in a single dose [8]. Despite the FDA approval of IN nalmefene, there is a lack of published evidence regarding its utilization, namely regarding concerns about its potential for precipitate withdrawal.

In this case report, one (self-reported) opioid-dependent adult, who had an overdose reversal by an Oakland County Sheriff’s Office (OCSO) officer using IN nalmefene, was randomly selected with an electronic randomizer by the local non-profit organization Alliance of Coalitions for Healthy Communities (ACHC). Two physicians, one board-certified in Addiction Medicine, the other in Emergency Medicine, independently reviewed OCSO bodycam footage of the overdose reversal, the associated dispatch report and patient interview data conducted 72 hours post-overdose by ACHC. The physicians conducted their clinical assessment independently of each other and combined their analyses for a joint case report to describe clinical signs and symptoms before, during, and after intranasal nalmefene administration.

## Case presentation

Case 1.1 (Investigator 1)

EMS and a sheriff’s officer arrived at the scene in response to a call of an unconscious but breathing male “who took a bunch of pills.” The 48-year-old white male was found supine, unconscious, and unresponsive with shallow respirations and reported pinpoint pupils. He did not respond to sternal rubbing. His girlfriend was present on scene and reported that she had found the patient unresponsive in the first-floor restroom, laid him on the floor outside the restroom and called for help. She believed he had consumed alcohol that day. She also reported that he had recently been seen in an ER for a bleeding ear. During the encounter, a straw with white powder residue was found in the waste basket of the first-floor restroom.

The first dose of IN nalmefene (2.7 mg in a single-use device) was given about 30 seconds into the video. Approximately 2 minutes later he was observed to be breathing more deeply with pulse-oximetry 88%. His pulse-oximetry increased to 98% approximately 1 minute later and the patient continued with improved spontaneous breathing.

Around 4 ½ minutes after nalmefene administration, the patient tried to sit up and was moving his hands. The second dose of nalmefene was given 5 ½ minutes after the initial dose. At the time of transport from the scene the patient was breathing well and continued to improve with a stable blood pressure (BP) of 110/78. He was awake but remained supine, trying to get up. There were no signs of withdrawal observed at any time following opioid reversal.

During the follow-up interview, the patient reported no negative side effects after the reversal. He admitted to using prescription medications for quite some time to cope with life stressors. He stated that on the evening of the overdose he had severe pain from his ear infection, and he bought some pills from an unfamiliar source.

Case 1.2 (Investigator 2)

A 48-year-old white male was found lying supine on the floor with his girlfriend in the room. She reported that she found the patient unresponsive in the first-floor bathroom and moved him to the area where he was found. She reported that he had possibly been drinking alcohol and shared that he had been seen in a local Emergency Room for bleeding from his ear in the last few days. While on scene, a straw with white powder in it was found in the bathroom trash can.

With the start of video at T 00:00 the patient was reported at T 00:24 to have a Glasgow Coma Scale (GCS) of 3 with no response to sternal rub. The patient was observed to have spontaneous but shallow sonorous respirations at what appeared to be at least a normal rate. The first dose of IN nalmefene was given at T 00:34. At T 01:22 there was still no response to sternal rub and the attendant stated “55 more seconds for the second” dose. A pulse-oximeter was placed at T 02:03 and patient was reported to still have pinpoint pupils. Oxygen saturation at T 02:22 was 88%. “Breaths are getting bigger” reported at T 02:28 and oxygen saturation increased to 98% at T 02:35. Seven seconds later the patient opened his eyes slightly and moved in response to sternal rub. The patient was reported to be moving his eyes at T 03:48 (GCS=6). Blood pressure at T 04:04 was 110/78. Sternal rub at T 05:04 (4 minutes and 30 seconds after IN nalmefene dose) caused patient to move his arm and attempt to sit up (GCS=9). The Sheriff's officer reported at T 05:44 that she did not repeat IN nalmefene because the patient was “still breathing” but then gave a second dose at T 06:06 with no observed change in respirations. The patient was then loaded into the ambulance without further incident.

Follow-up documentation about one month later states that the patient reported “no negative side effects after being revived…and felt fine”. He did eventually admit to “using prescription pills for some time” to deal with life stressors. The patient also revealed that he had “received pills from a source that he’s not used to” to treat severe ear pain.

## Discussion

The widespread availability of synthetic opioids has contributed to fentanyl-related deaths rising 29-fold from 2012 to 2020 [9]. Potent, long-acting synthetic opioids require higher and repeated naloxone doses. Higher potency synthetic opioids (e.g., carfentanil) and opioid combinations (e.g., fentanyl and carfentanil with heroin), which may be less responsive to naloxone, are of increasing concern [10].

With a higher affinity for opioid receptors and a longer duration of action compared to naloxone, IN nalmefene is a promising alternative opioid overdose treatment [11-13]. IN nalmefene rapidly reverses opioid-induced respiratory depression compared to IN naloxone [8] and in a validated model of simulated synthetic opioid overdoses, IN nalmefene was more effective than IN naloxone in reducing the incidence of cardiac arrest following potentially lethal doses of both fentanyl and carfentanil [14]. Its longer half-life (11 hours) may also reduce renarcotization events compared to naloxone (1.1 to 2 hours). According to the prescribing information of IN nalmefene, if the initial dose is ineffective, a second dose may be administered after a two-minute interval [4]. The required amount of opioid antagonist can vary depending on several factors, including the specific opioid involved, the route of administration, and patient tolerance.

In this case study, bodycam footage of opioid overdose reversal using IN nalmefene was reviewed. It is noted that improvement in respirations with an increase in oxygen saturation from 88% to 98% occurred after the initial dose. The patient also exhibited a marked improvement in mental status, going from no response with a prolonged deep sternal rub to attempting to sit up with movement of his arms. His blood pressure was normal. The investigators note that a second dose may have been unnecessary as breathing was already restored. Notably, the patient did not demonstrate any signs of precipitated opioid withdrawal (e.g., gastrointestinal distress, dilated pupils, tachycardia, diaphoresis, rhinorrhea, yawning, aches, anxiety, and restlessness).

## Conclusions

This report indicates that IN nalmefene can rapidly reverse an opioid overdose. No evidence of precipitated withdrawal was noted. The rapid clinical response and the potential for a reduced need for repeat dosing have important implications for treatment in the field and at the receiving medical facility. Limitations include reliance on a single twelve-minute bodycam footage from one patient for clinical evaluation and a lack of toxicological and emergency department information. Future studies are important to establish IN nalmefene’s impact on clinical outcomes once patients arrive at accepting medical facilities.
